# Knowledge and Attitude Related to Hepatitis C among Medical Students in the Oral Direct Acting Antiviral Agents Era in Vietnam

**DOI:** 10.3390/ijerph191912298

**Published:** 2022-09-28

**Authors:** Thi Thanh Hang Pham, Thi Thuy Linh Nguyen, Samuel So, Thi Hai Van Hoang, Thi To Uyen Nguyen, Thanh Binh Ngo, Minh Phuong Nguyen, Quang Hung Thai, Ngoc Khoi Nguyen, Thi Quynh Anh Le Ho, Quang Phuc Tran, Trung Son Mai, Mehlika Toy, Minh Khue Pham

**Affiliations:** 1 Asian Liver Center, Department of Surgery, Stanford University School of Medicine, Palo Alto, CA 94304, USA; 2 Faculty of Public Health, Haiphong University of Medicine and Pharmacy, Haiphong 04212, Vietnam; 3 Department of Global Health, Hanoi Medical University, Hanoi 11521, Vietnam; 4 Faculty of Public Health, Thai Nguyen University of Medicine and Pharmacy, Thai Nguyen 24117, Vietnam; 5 Department of Oto-Rhino-Laryngology, Thai Binh University of Medicine and Pharmacy, Thai Binh 06118, Vietnam; 6 Department of Pediatrics, Can Tho University of Medicine and Pharmacy, Can Tho 94117, Vietnam; 7 Department of Public Health, Faculty of Medicine and Pharmacy, Tay Nguyen University, Dak Lak 63000, Vietnam; 8 Department of Clinical Pharmacy, University of Medicine and Pharmacy at Ho Chi Minh City, Ho Chi Minh City 71006, Vietnam; 9 Family Medicine Center, Hue University of Medicine and Pharmacy, Hue University, Hue 49000, Vietnam; 10 General Office for Population and Family Planning, Vietnam Ministry of Health, Hanoi 12014, Vietnam

**Keywords:** HCV, knowledge, medical students, Vietnam

## Abstract

Background: Medical students play important frontline roles in the prevention, early detection, and treatment of hepatitis C. This study investigated knowledge and attitudes toward hepatitis C among 5th- and 6th-year medical students and possible associated factors. Methods: A cross-sectional survey was conducted among 2000 students from eight medical universities using a self-administered structured questionnaire. Results: The mean knowledge and attitude scores for hepatitis C were 20.1 ± 4.0 (out of 26) and 10.6 ± 2.9 (out of 20), respectively. Approximately, three-quarters (74.4%) of the participants had a good knowledge score, but only a small proportion (3.1%) obtained a good attitude score. Although the participants had fairly high knowledge about the causes, consequences, and transmission routes of hepatitis C, there were important gaps in their knowledge about hepatitis C screening and treatment. In multivariate analysis, female students, 5th-year students, and students from the central provinces had significantly higher knowledge and attitude scores. There was a low positive correlation between knowledge and attitude scores. Conclusion: This study points out the need to update the medical training curriculum to improve the knowledge and attitude of students about hepatitis C infection.

## 1. Introduction

The hepatitis C virus (HCV), which can cause both acute and chronic hepatitis, is estimated to affect 71 million people worldwide [1]. It is a leading cause of cirrhosis that can ultimately result in end-stage liver disease, hepatocellular carcinoma, and liver transplantation. It is estimated that approximately 500,000 people die from hepatitis C each year, mostly from cirrhosis and hepatocellular carcinoma, creating a substantial burden on the healthcare system [2]. The development of direct-acting antivirals (DAAs), which improve treatment outcomes significantly with minimal side effects and short treatment duration, has changed the HCV therapeutic landscape significantly in the past decade [3]. However, access to screening and treatment is limited, particularly in resource-limited settings.

In Vietnam, available data suggest that the seroprevalence of HCV infection is approximately 1–4% in the general population [4], but is substantially higher among HIV patients (22.9–89.0%), injecting drug users (74.0–87.0%), men who have sex with men (28.4–38.8%), and multitransfusion, and dialysis patients (6.0–26.6%) [5,6,7,8,9,10,11]. In the past, treatment for HCV infection in Vietnam was limited to interferon-based (IFN-gamma and pegylated-IFN) therapy. Newer DAA agent regimes have been introduced in Vietnam since 2016 and were added for the first time to the list of medicines partially reimbursable through health insurance in 2019.

Early diagnosis and access to treatment are essential components of an effective HCV prevention and control program. In Vietnam, the current guidelines for hepatitis C testing recommend screening for high-risk groups, including HIV patients, injecting drug users, men who have sex with men, sex workers, blood donors, and patients who need hemodialysis, immune-suppression therapies, or have abnormal ALT levels [12]. In settings with high HCV prevalence in the general population (defined as >2% or >5% HCV antibody seroprevalence), it is recommended by the WHO that all adults have access to and be offered HCV testing with linkage to prevention, care, and treatment services [1]. Therefore, healthcare workers and medical students play important frontline roles in HCV prevention, detection, and counseling. This study sought to evaluate medical students’ knowledge and attitudes toward HCV prevention and care to identify gaps and opportunities for improving the capacity of medical students in HCV prevention, testing, and management.

## 2. Materials and Methods

From May to November 2020, we conducted a cross-sectional survey at eight representative medical universities located in the northern, central, and southern regions of Vietnam.

### 2.1. Population and Sampling

Students in their last two years (5th or 6th) of their medical studies were eligible to participate in this study, as in their training program, they had completed the hepatology, infectious diseases, and epidemiology courses.

Our sample size calculation was based on the precision of the estimated prevalence of students having good knowledge and attitudes related to hepatitis B and C. As we do not have any prior data on this pattern, 50% was chosen as the default prevalence for sample size calculation, with a precision of ±5%. This gave us a minimum sample size estimation of 384 participants for this survey. Nevertheless, we wanted to recruit a number of students that were representative of all 8 medical universities in the country. Each reportedly had approximately 300–500 students in their 5th or 6th year. Therefore, from the consensus of all medical universities, we recruited 250 students from each university, which makes 2000 participants in total. This number is big enough to satisfy the minimum sample size calculated above.

The study recruited 2000 students using a systematic random sampling technique to evaluate their knowledge, attitudes, and practices regarding hepatitis B and C. The recruitment process and the results on knowledge, attitudes, and practices towards the hepatitis B virus infection among these medical students were published elsewhere [13]. This report summarizes the results on knowledge and attitudes of medical students on hepatitis C prevention and care.

### 2.2. Questionnaire

A self-administered questionnaire was developed based on a review of existing tools used to evaluate HCV knowledge, attitudes, and practice of medical students and healthcare workers [14,15,16,17,18]. The predesigned questionnaire was tested on a random sample of students (n = 20) to ensure understandability and clarity of the questions before distribution. There were 26 questions on HCV knowledge and 4 questions on HCV attitudes. The demographic characteristics included in this study are age, gender, and school year (See Supplementary File S1).

### 2.3. Definitions for Scoring Knowledge and Attitudes

Knowledge of the study participants was assessed by 26 questions (C61–C86).

There were five questions on cause and consequences, eight questions on transmission routes, six questions on prevention and screening, and seven questions on treatment and monitoring. Each correct knowledge answer was given 1 point. Incorrect or “don’t know” answers received 0 points.

Attitudes of the study participants were assessed by 4 questions (C87–C90) with the “strongly disagree” answer having the highest knowledge (4 points) and the “strongly agree” answer having the lowest score (0 points).

In this study, a modified Bloom’s cutoff value was used to categorize participants’ knowledge and attitudes [19,20,21,22]. A participant was considered to have a good score if the individual obtained at least 75% of the maximum score. Participants who scored less than 75% of the maximum score were considered to have poor scores.

### 2.4. Data Collection and Analysis

Data were entered into EpiData 3.1 (EpiData Association, Odense, Denmark), and then imported and analyzed using RStudio and Excel. Descriptive statistics (frequency, mean, and SD) were used for each knowledge and attitude question. Knowledge scores and attitude scores were used as the primary practice outcome variables for examining potential predictive factors. Univariate logistic regression was performed to examine the association between outcome variables and potential predictor variables, including gender, school year, and region. Variables with a *p*-value < 0.25 in univariate were included in the multivariate analysis. Regression coefficients and their 95% confidence intervals (CIs) were computed to measure the relative importance of each independent variable on the outcome variable.

The degree of statistical significance was established at a *p*-value ≤ 0.05.

### 2.5. Ethical Consideration

The study was approved by the Research and Ethics Committee at Haiphong University of Medicine and Pharmacy. Written informed consent was obtained from all participants prior to the survey. No personal identifying information was collected to ensure confidentiality.

## 3. Results

### 3.1. Knowledge about Hepatitis C Prevention, Screening, and Treatment

All 2000 students who were invited to participate in the study returned completed questionnaires, resulting in 100% response rate. Approximately, 54.4% of them were female and 45.5% were male. The mean age of the study participants was 23.7 ± 0.9 years (range: 21–30 years). More students in their 5th medical school year (1250 or 62.5%) participated than those in their 6th year (750 or 37.5%).

Table 1 summarizes the frequency and percentage of correct answers for each knowledge question. The majority of the participants knew that HCV infection could lead to liver cirrhosis (92.8%) and that it increased the risk of developing liver cancer (89.6%). However, 15.6% of the study participants mistakenly believed that hepatitis C was a mutation of the hepatitis B virus.

The surveyed participants had good knowledge about the transmission routes of HCV. Most of them provided correct answers that HCV can be transmitted through sharing injection equipment (96.7%), blood-to-blood contact (91.8%), and blood transfusion (2%). However, 5% wrongly believed that HCV could be transmitted through casual contact, such as kissing or hugging. Approximately, one-tenth (10.1%) incorrectly believed that HCV was airborne in an enclosed environment. More than half (58.2%) thought that people with hepatitis C should be restricted from working in the food industry. Approximately, 31.3% mistakenly thought that sexual transmission was the most common cause of hepatitis C. Approximately, 38.5% thought that HCV is vaccine-preventable, and 38.5% believed that individuals who were infected before with hepatitis C would not be reinfected. Approximately, 44.7% falsely thought that HIV was more contagious through blood contact than HCV.

Most of the participants knew that people with HCV infection may not present any symptoms (86.2%) and they should limit alcohol intake (92.4%). However, knowledge about other aspects of HCV treatment is limited. Approximately, a third of the study participants (31.3%) did not know that there are medications to treat hepatitis C and that HCV infection is curable. Approximately, 22.2% of the study participants were not aware of the availability of DAA medications for HCV treatment. Approximately, a third (35.9%) did not know that the average treatment duration of DAAs is approximately 12 weeks.

The mean values for the total knowledge score and each subcategory are shown in Table 2. The overall mean knowledge score was 20.11 out of 26 (77.4%), indicating a relatively good level of knowledge about HCV among the study participants. Approximately one-fourth (74.4%) of the students surveyed had a good knowledge score, which was defined as having a score of at least 70% of the maximum score. The mean knowledge score for prevention and screening was the lowest (3.9 out of 6, equivalent to 64.6%). The highest knowledge mean scores were 4.6 out of 5 (91.3%) for cause and consequences, followed by knowledge mean scores for transmission routes at 6.46 out of 8 (80.7%).

Regression analysis was conducted to examine the relationship between knowledge scores and potential predictors, including gender, school year, and region in Table 3. The results established that female students, 5th-year students, and students from the southern and central regions had significantly higher knowledge scores.

### 3.2. Attitude toward HCV Care and Management

Attitudes toward HCV patients were evaluated by 5-point Likert-scale questions in Table 4, with “strongly disagree” having the highest knowledge (4 points) and “strongly agree” receiving the lowest score (0 point). Approximately 64.1% strongly agreed or agreed that all patients should be tested for HCV before receiving healthcare. As many as 91.8% of the study participants strongly agreed or agreed that the HCV status of patients should be disclosed in healthcare settings for safety reasons. More than a fourth (27.5%) strongly agreed or agreed that HCV patients be scheduled for the last appointment of the day.

Overall, the mean attitude score was 10.6 out of 16 (66.4%), with only 63 participants (3.2%) having a good attitude score. In multivariate linear regression analysis, female students, 5th-year students, and those from the central and southern regions had significantly higher attitude scores. There was a weak positive relationship between knowledge scores and attitude scores (adjusted coefficient = 0.17, *p* < 0.001) (Table 5).

## 4. Discussion

The overall knowledge level of the medical students about HCV infection in the present study is fairly high, with 74.4% having a good knowledge score. In a survey among fourth-year medical students by Almansour et al. in Saudi Arabia [14], the HCV knowledge level was lower with 30.3% having a poor knowledge score (<50% of the maximum score) and 69.7% having a fair knowledge score (50–75% of the maximum score). Similarly, Khan et al. reported a lower HCV knowledge score in which between 50% and 70% of the medical student participants in Pakistan were in the “good” category [16].

The participants in our study had good knowledge about HCV transmission routes, with more than 90% being aware of HCV transmission through sharing injection equipment, blood-to-blood contact, and blood transfusion. Those results are higher than previously reported in studies among medical students in Saudi Arabia [14], Pakistan [16], Iran [23], and Syria [17]. The majority of the students in our study knew that a patient with hepatitis C can be asymptomatic (86.2%) and can lead to cirrhosis or liver cancer (>90%). Similar findings were reported in medical students in Saudi Arabia [14].

Our study revealed important knowledge gaps pertaining to HCV prevention, screening, and treatment. For example, 38.5% of the study participants were not aware that there is currently no vaccine to prevent HCV infection. A third (33.4%) of the participants failed to understand that an individual could have hepatitis C antibodies even though they are not currently infected with the virus. Approximately a third of the study participants did not know that there are medications for hepatitis C treatment and that HCV infection is curable. Awareness about the availability of DAAs therapy in treating HCV and the treatment duration was insufficient. This particular knowledge is essential to prepare medical students to engage in HCV prevention, screening, referral to specialists, and potentially treating HCV patients. In healthcare settings, universal precaution measures will be practiced in such a way that the blood and certain body fluids of all patients must be considered potentially infectious for HIV, HCV, and other blood-borne pathogens. In other words, healthcare workers need to treat all potentially infectious materials as if they are infected. In this study, a surprisingly high proportion of the participants believed that patients’ HCV status should be disclosed for safety reasons in healthcare settings (91.8%), all patients should be tested for HCV before receiving healthcare (64.1%), and HCV-infected patients should be scheduled for the last appointment of the day (27.5%). Similar findings were reported in Iran by Mansour-Ghanaei et al. in which 95.8% agreed that HCV patients should be identified for infection control purposes, 88.3% agreed that all patients should be tested for HCV before receiving healthcare, and 29.2% agreed that patients with HCV should be given the last appointment for the day [23]. This could partially be explained by the students’ misconceptions that HCV could be transmitted through casual contacts (5.0%) or airborne (10.2%). While the present study did not specifically assess participants’ willingness to treat patients with HCV, negative attitudes among medical students could potentially lead to discriminatory practices, such as denial of care or excessive precautions toward HCV patients.

Our study results indicated a low positive correlation between medical students’ knowledge and attitude scores. This is in line with results from similar studies of medical students and healthcare workers [15,23,24]. However, a study by Mortel et al. showed no significant correlation between HCV knowledge and attitude among healthcare workers [18]. In this study, there was a significant association between gender and mean knowledge score in which female students scored higher than male students (adjusted Coeff. = 0.57, *p* < 0.001). However, the association between gender and the mean HCV knowledge score was not significant in medical students in Saudi Arabia [14] and Iran [23]. In this study, there was a significant difference in knowledge and attitude scores between the school years, with students in the fifth year having higher scores than those in the sixth year. It is possible that the 5th year students had just finished training modules on hepatology, infectious diseases, and epidemiology prior to participating in the survey, so they had more recent knowledge acquisition about HCV. In a study about the knowledge and attitudes of medical students about HBV, students in the fifth year also had higher scores than those in the sixth year [13]. To address this challenge, it is critical that medical universities apply a competency-based training approach, which will not only help students acquire knowledge but also evaluate how they apply knowledge to clinical situations that physicians often face in order to sustain that knowledge and skills after graduation.

Based on the knowledge gaps pertaining to HCV prevention, screening, and treatment, it is important to focus on including viral hepatitis education in medical schools and healthcare staff in hospitals in general. The disease knowledge, as well as the treatment and prevention intervention knowledge, should be included in the medical school curriculum. The results from this study can help address this gap in the curriculum and can be used to make a case to educate medical students and healthcare staff on viral hepatitis interventions and disease knowledge. In return, by adding this kind of education to the curriculum, viral hepatitis in Vietnam can be tackled smoothly. Since the attitudes of the medical students in this study toward HCV are negative and can influence the quality of services for patients with HCV, the national recommendations can focus on addressing this gap by simplifying the material and making sure that these recommendations are thought in medical school and repeated for healthcare staff that see HCV patients in clinics.

This study enrolled a large sample of participants (n = 2000, accounting for approximately 50% of all 5th and 6th year students) from all eight medical universities in the northern, central, and southern regions of Vietnam with the highest response rate (100%). As such, the representativeness of the findings was very high. The data in our study, however, has limitations because it was based on self-reported responses which could not be validated. In addition, the study was cross-sectional in design, and therefore, a causal relationship could not be established.

## 5. Conclusions

Even though the study participants’ overall level of knowledge was fairly high, there were important knowledge and attitude gaps pertaining to HCV prevention, screening, and management that need to be properly addressed in medical education programs. In general, attitudes of the medical students in this study toward HCV are negative and could potentially influence the accessibility and quality of services for patients with HCV.

## Figures and Tables

**Table 1:** Correct knowledge answers toward HCV (n = 2000).

TOTAL: 26 Questions	Correct Answer	
	N	%
HCV cause and consequences (n = 5)		
Q61. Is Hepatitis C caused by a virus?	1915	95.8%
Q62. Is Hepatitis C caused by bacteria?	1881	94.1%
Q72. Can hepatitis C develop into cirrhosis?	1856	92.8%
Q73. Does hepatitis C increase your risk of liver cancer?	1791	89.6%
Q74. Is hepatitis C a mutation of hepatitis B?	1689	84.5%
Transmission Routes (n = 8)		
Q63. Can hepatitis C spread through casual contacts, such as kissing or hugging?	1501	75.1%
Q64. Can hepatitis C be spread through sharing injection equipment, such as needles, syringes, or medicine spoons?	1839	2.0%
Q65. Can mosquitoes be a vector for hepatitis C?	1625	81.3%
Q66. Can Hepatitis C spread through blood-to-blood contact?	1836	91.8%
Q67. Is hepatitis C airborne in an enclosed environment (for example, crowded buses and lifts)?	1798	89.9%
Q68. Is sexual transmission the most common cause of hepatitis C?	625	31.3%
Q69. People can get hepatitis C through nonsterilized equipment when getting a tattoo.	1850	92.5%
Q70. People can get hepatitis C through a blood transfusion.	1839	2.0%
Treatment and Monitoring (n = 7)		
Q75. Can someone get hepatitis C and not have any symptoms?	1723	86.2%
Q76. Are there currently medications to treat hepatitis C?	1373	68.7%
Q77. Can Hepatitis C be cured?	1373	68.7%
Q81. Should people with hepatitis C limit alcohol intake?	1847	92.4%
Q84. The current treatment regimen for chronic hepatitis C is a direct acting antiviral (DAAs)?	1555	77.8%
Q85. Oral medication (DAAs) treatment regimen have an average treatment duration of 12 weeks	1281	64.1%
Q86. Treatment response period for hepatitis C is when the patient achieves a sustained viral response 12 weeks after the end of treatment (SVR12).	1272	63.6%
Prevention and Screening (n = 6)		
Q71. Must people with hepatitis C be restricted from working in the food industry?	1163	58.2%
Q78. Is there a vaccine to prevent Hepatitis C infection?	1231	61.6%
Q79. Is HIV more infectious in blood-to-blood contact than Hepatitis C?	1105	55.3%
Q80. Can an individual have hepatitis C antibodies even though they are not currently infected with the virus?	1352	67.6%
Q82. If you have been infected with hepatitis C, can you be reinfected?	1229	61.5%
Q83. If the hepatitis C virus stays in the blood and liver for more than 6 months after infection, is chronic hepatitis C diagnosed?	1668	83.4%

**Table 2:** Mean knowledge scores (n = 2000).

Knowledge Subcategories	Number of Questions	Mean	SD (Range)	Percentage to Maximum Score
HCV cause and consequences	5	4.6	0.9	91.3%
Transmission Routes	8	6.5	1.5	80.7%
Prevention and Screening	6	3.9	1.4	64.6%
Treatment and Monitoring	7	5.2	1.7	74.5%
TOTAL KNOWLEDGE SCORE	26	20.1	4.0	77.4%

**Table 3:** Predictors of HCV knowledge scores (n = 2000).

	Univariate Linear Regression Analysis			Multivariate Linear Regression Analysis		
	Coeff.	t	*p*	Adjusted Coeff.	95% CI	*p*
Gender						
Male (reference)						
Female	0.73	4.09	<0.001	0.94	0.60 to 1.28	<0.001
Region						
North (reference)						
Central	2.51	9.14	<0.001	2.13	1.59 to 2.67	<0.001
South	0.8	4.27	<0.001	1.7	1.30 to 2.10	<0.001
School year						
5th (reference)						
6th	−1.47	−8.06	<0.001	−1.85	−2.45 to −1.45	<0.001

**Table 4:** Students’ attitudes toward HCV (n = 2000).

Questions	N	%
Q87. In healthcare settings, should the HCV status of patients be disclosed for safety reasons?		
A. Strongly Agree	1054	52.7%
B. Agree	782	39.1%
C. Neutral	84	4.2%
D. Disagree	68	3.4%
E. Strongly Disagree	12	0.6%
Q88. Should HCV-infected patients be scheduled for the last appointment of the day?		
A. Strongly Agree	225	11.3%
B. Agree	324	16.2%
C. Neutral	354	17.7%
D. Disagree	873	43.7%
E. Strongly Disagree	224	11.2%
Q89. Should healthcare workers with HCV infection avoid contact with patients?		
A. Strongly Agree	182	9.1%
B. Agree	315	15.8%
C. Neutral	300	15%
D. Disagree	923	46.2%
E. Strongly Disagree	280	14%
Q90. Should all patients be tested for HCV before receiving health care?		
A. Strongly Agree	490	24.5%
B. Agree	791	39.6%
C. Neutral	317	15.9%
D. Disagree	349	17.5%
E. Strongly Disagree	53	2.7%

**Table 5:** Predictors of attitude scores (n = 2000).

	Univariate Linear Regression Analysis			Multivariate Linear Regression Analysis		
	Coeff.	T	*p*	Adjusted Coeff.	95% CI	*p*
Gender						
Male (reference)						
Female	0.34	2.63	0.009	0.38	0.14 to 0.63	0.01
Region						
North (reference)						
Central	1.66	−8.29	<0.001	1.06	0.67 to 1.45	<0.001
South	1.11	−8.33	<0.001	1.38	1.09 to 1.67	<0.001
School year						
5th (reference)						
6th	−0.59	−4.47	<0.001	−0.79	−1.08 to −0.50	<0.001
Knowledge score	0.21	13.5	<0.001	0.17	0.14 to 0.20	<0.001

## Data Availability

The STATA data used to support the findings of this study are available from the corresponding author upon request.

## Supplementary Materials

The following are available online at https://www.mdpi.com/article/10.3390/ijerph191912298/s1, Supplementary File S1: Questionnaire of the survey.
