# Supplementary material for: Knowledge and Attitude Related to Hepatitis C among Medical Students in the Oral Direct Acting Antiviral Agents Era in Vietnam
Source: Int J Environ Res Public Health. 2022 Sep 28;19(19):12298. doi: 10.3390/ijerph191912298 (PMC9565151; doi:10.3390/ijerph191912298)
Supplement: Supplementary file 1 [file ijerph-19-12298-s001.zip › ijerph-1787017-supplementary.pdf]

STROBE (Strengthening The Reporting of OBservational Studies in Epidemiology) Checklist

A checklist of items that should be included in reports of observational studies. You must report the page number in your manuscript where you consider each of the items listed in this checklist. If you have not included this information, either revise your manuscript accordingly before submitting or note N/A.

Note: An Explanation and Elaboration article discusses each checklist item and gives methodological background and published examples of transparent reporting. The STROBE checklist is best used in conjunction with this article (freely available on the Web sites of PLoS Medicine at http://www.plosmedicine.org/, Annals of Internal Medicine at http://www.annals.org/, and Epidemiology at http://www.epidem.com/). Information on the STROBE Initiative is available at www.strobe-statement.org.

Section and Item	Item No.	Recommendation	Reported on Page No.
**Title and Abstract**	1	(*a*) Indicate the study's design with a commonly used term in the title or the abstract	page 1 (line 31)
		(*b*) Provide in the abstract an informative and balanced summary of what was done and what was found	page 1 (line 32-39)
**Introduction**			
Background/Rationale	2	Explain the scientific background and rationale for the investigation being reported	page 2 (line 46-72)
Objectives	3	State specific objectives, including any prespecified hypotheses	page 2 (line 73-76)
**Methods**			
Study Design	4	Present key elements of study design early in the paper	page 2 (line 78-79)
Setting	5	Describe the setting, locations, and relevant dates, including periods of recruitment, exposure, follow-up, and data collection	page 2-3 (line 78-84; 94-96; 121-131)
Participants	6	(*a*) *Cohort study*—Give the eligibility criteria, and the sources and methods of selection of participants. Describe methods of follow-up	
		Case-control study—Give the eligibility criteria, and the sources and methods of case ascertainment and control selection. Give the rationale for the choice of cases and controls	
		Cross-sectional study—Give the eligibility criteria, and the sources and methods of selection of participants	page 2 (line 82-99)
		(*b*) *Cohort study*—For matched studies, give matching criteria and number of exposed and unexposed	
		Case-control study—For matched studies, give matching criteria and the number of controls per case	
Variables	7	Clearly define all outcomes, exposures, predictors, potential confounders, and effect modifiers. Give diagnostic criteria, if applicable	page 3 (line 105-120)

Section and Item	Item No.	Recommendation	Reported on Page No.
Data Sources/ Measurement	8*	For each variable of interest, give sources of data and details of methods of assessment (measurement). Describe comparability of assessment methods if there is more than one group	page 3 (line 108-120)
Bias	9	Describe any efforts to address potential sources of bias	page 3 (line 103-104)
Study Size	10	Explain how the study size was arrived at	page 2 (line 94-96)
Quantitative Variables	11	Explain how quantitative variables were handled in the analyses. If applicable, describe which groupings were chosen and why	page 3 (line 108-120)
Statistical Methods	12	(*a*) Describe all statistical methods, including those used to control for confounding	page 3 (line 121-131)
		(*b*) Describe any methods used to examine subgroups and interactions	page 3 (line 121-131)
		(*c*) Explain how missing data were addressed	page 3 (line 121-131)
		(*d*) *Cohort study*—If applicable, explain how loss to follow-up was addressed	
		Case-control study—If applicable, explain how matching of cases and controls was addressed	
		Cross-sectional study—If applicable, describe analytical methods taking account of sampling strategy	page 3 (line 121-131)
		(*e*) Describe any sensitivity analyses	
**Results**			
Participants	13*	(a) Report numbers of individuals at each stage of study—eg numbers potentially eligible, examined for eligibility, confirmed eligible, included in the study, completing follow-up, and analysed	page 3 (line 139)
		(b) Give reasons for non-participation at each stage	page 3 (line 140)
		(c) Consider use of a flow diagram	
Descriptive Data	14*	(a) Give characteristics of study participants (eg demographic, clinical, social) and information on exposures and potential confounders	page 3 (line 141-143)
		(b) Indicate number of participants with missing data for each variable of interest	NA
		(c) *Cohort study*—Summarise follow-up time (eg, average and total amount)	
Outcome Data	15*	*Cohort study*—Report numbers of outcome events or summary measures over time	
		Case-control study—Report numbers in each exposure category, or summary measures of exposure	
		Cross-sectional study—Report numbers of outcome events or summary measures	page 3 (line 144-147)

Section and Item	Item No.	Recommendation	Reported on Page No.
Main Results	16	(*a*) Give unadjusted estimates and, if applicable, confounder-adjusted estimates and their precision (eg, 95% confidence interval). Make clear which confounders were adjusted for and why they were included	page 4 (line 149-167)
		(*b*) Report category boundaries when continuous variables were categorized	page 5 (line 176-180)
		(*c*) If relevant, consider translating estimates of relative risk into absolute risk for a meaningful time period	page 5 (line 181-189; 196-197)
Other Analyses	17	Report other analyses done—eg analyses of subgroups and interactions, and sensitivity analyses	page 6 (line 196-197)
**Discussion**			
Key Results	18	Summarise key results with reference to study objectives	page 6- 7 (line 198-211)
Limitations	19	Discuss limitations of the study, taking into account sources of potential bias or imprecision. Discuss both direction and magnitude of any potential bias	page 8 (line 271-272)
Interpretation	20	Give a cautious overall interpretation of results considering objectives, limitations, multiplicity of analyses, results from similar studies, and other relevant evidence	page 8 (line 273-274)
Generalisability	21	Discuss the generalisability (external validity) of the study results	page 8 (line 270-271)
**Other Information**			
Funding	22	Give the source of funding and the role of the funders for the present study and, if applicable, for the original study on which the present article is based	page 8 (line 290-291)

*Give information separately for cases and controls in case-control studies and, if applicable, for exposed and unexposed groups in cohort and cross-sectional studies.

Once you have completed this checklist, please save a copy and upload it as part of your submission. DO NOT include this checklist as part of the main manuscript document. It must be uploaded as a separate file.
